# Síntesis de evidencia: directrices de práctica clínica basadas en la evidencia para el seguimiento de recién nacidos en riesgo

**DOI:** 10.26633/RPSP.2021.141

**Published:** 2021-12-22

**Authors:** Organización Panamericana de la Salud

**Affiliations:** 1 Organización Panamericana de la Salud Washington, D.C. Estados Unidos de América Organización Panamericana de la Salud, Washington, D.C., Estados Unidos de América.

**Keywords:** Neonatología, recien nacido prematuro, medicina basada en la evidencia, Américas, Neonatology, Infant, premature, evidence-based medicine, Americas, Neonatologia, recém-nascido prematuro, medicina baseada em evidências, América

## Abstract

**Introducción.:**

La Organización Mundial de la Salud recomienda focalizar la atención en el período neonatal, eliminar la mortalidad de causa prevenible y brindar cuidados de calidad. Es esencial conocer cuáles son las condiciones con alta probabilidad de ocurrencia en esa población para monitorearlos de forma sistemática, de modo que se logre su detección temprana; y el abordaje terapéutico y rehabilitación oportunos.

**Objetivos.:**

Sintetizar las recomendaciones incluidas en las *Directrices de práctica clínica basadas en la evidencia para el seguimiento de recién nacidos en riesgo*, publicada por la Centro Latinoamericano de Peri­natología/Salud de la Mujer y Reproductiva de la Organización Panamericana de la Salud en el 2020, con el fin de presentar las estrategias para el seguimiento de los niños recién nacidos con condiciones de riesgo desde su nacimiento hasta los 2 años.

**Métodos.:**

Se llevó a cabo una síntesis de la guía y sus recomendaciones. Además, se realizó una búsqueda sistemática en Pubmed, Lilacs, Health Systems Evidence, Epistemonikos y literatura gris de estudios desarrollados en la Región de las Américas, con el fin de identificar barreras, facilitadores y estrategias de implementación.

**Resultados.:**

Se formularon 21 recomendaciones y 14 puntos de buena práctica que aplican a los recién nacidos con condiciones de riesgo hasta los dos años (prematuros y aquellos con alteraciones adquiridas o congénitas). Se identificaron barreras como la disponibilidad de pruebas de tamización, deficiencias en el sistema de referencia y conocimiento de las recomendaciones para su implementación.

**Conclusiones.:**

La guía brinda recomendaciones sobre los criterios de egreso, incluidas pruebas de tamizaje; información y apoyo para padres y cuidadores; tamizaje y frecuencia de seguimiento de los niños en riesgo hasta los dos años en la Región de las Américas.

## Abstract (en)

**Introduction.:**

The World Health Organization recommends focusing attention on the neonatal period, eliminating mortality from preventable causes, and providing quality care. It is essential to know which conditions have a high probability of occurring in that population in order to monitor them systematically, detect them early, and provide timely treatment and rehabilitation.

**Objectives.:**

Synthesize the recommendations contained in *Directrices de práctica clínica basadas en la evidencia para el seguimiento de recién nacidos en riesgo* [Evidence-based Clinical Practice Guidelines for Monitoring At-risk Newborns], published in Spanish in 2020 by the Pan American Health Organization’s Latin American Center for Perinatology, Women's Health, and Reproductive Health, with a view to producing strategies for monitoring at-risk newborns from birth up to the age of 2 years.

**Methods.:**

The guidelines and recommendations were synthesized and a systematic search was conducted in PubMed, Lilacs, Health Systems Evidence, Epistemonikos, and gray literature for studies carried out in the Region of the Americas with a view to identifying barriers, facilitators, and implementation strategies.

**Results.:**

A total of 21 recommendations and 14 good practices were formulated for application to newborns with at-risk conditions (prematurity and congenital or acquired conditions) up to 2 years of age. The barriers identified as standing in the way of their implementation were insufficient availability of screening tests, deficiencies in the referral system, and lack of awareness of the recommendations.

**Conclusions.:**

The guidelines include recommendations for discharge criteria, screening, and frequency of monitoring for at-risk newborns up to 2 years of age in the Region of the Americas, as well as information and support for parents and caregivers.

## RESUMO

**Introdução.:**

A Organização Mundial da Saúde recomenda focar a atenção no período neonatal, eliminar a mortalidade decorrente de causas preveníveis e oferecer cuidados de qualidade. É essencial conhecer quais são as condições com alta probabilidade de ocorrência nessa população para monitorá-la de forma sistemática, de maneira que ocorra uma detecção precoce, assim como conhecer a abordagem terapêutica e a reabilitação adequadas.

**Objetivos.:**

Sintetizar as recomendações incluídas nas *Diretrizes de prática clínica baseada em evidência para o acompanhamento de recém-nascidos em risco*, publicadas em 2020 pelo Centro Latino-Americano de Perinatologia, Saúde da Mulher e Reprodutiva da Organização Pan-Americana da Saúde, com o propósito de apresentar estratégias para o acompanhamento de recém-nascidos em situação de risco, do nascimento até os 2 anos de idade.

**Métodos.:**

Foi realizada uma síntese do guia e de suas recomendações. Além disso, foi realizada uma busca sistemática nas bases Pubmed, Lilacs, Health Systems Evidence e Epistemonikos e na literatura cinzenta de estudos conduzidos na Região das Américas para identificar barreiras, facilitadores e estratégias de implementação.

**Resultados.:**

Foram formuladas 21 recomendações e 14 pontos de boas práticas que se aplicam aos recém-nascidos em situação de risco até os dois anos de idade (prematuros e aqueles com alterações adquiridas ou congênitas). Foram identificadas barreiras como a disponibilidade de testes de triagem, deficiências no sistema de encaminhamento e conhecimento das recomendações para sua implementação.

**Conclusões.:**

O guia oferece recomendações sobre os critérios de alta hospitalar, incluindo testes de triagem; informação e apoio para pais e cuidadores; e triagem e frequência de acompanhamento das crianças em risco até os dois anos de idade na Região das Américas.

## INTRODUCCIÓN

Los avances en el cuidado neonatal en el mundo, y particularmente en América Latina y el Caribe, han contribuido de manera sustantiva a la reducción en la mortalidad neonatal permitiendo el aumento de la supervivencia de recién nacidos prematuros o con condiciones que los exponen a riesgos que pueden incidir en su crecimiento, desarrollo y calidad de vida. Los resultados en la supervivencia, así como en la reducción del efecto de las condiciones específicas están vinculados tanto al cuidado del recién nacido en torno al nacimiento y en unidades de cuidados especiales como al seguimiento de los recién nacidos con tales condiciones (1,2).

Durante la estadía en las unidades de cuidados intensivos neonatales (UCIN), los niños con condiciones que los exponen a riesgos específicos requieren de cuidados especializados y seguimiento acorde a las condiciones de riesgo biológico o sociales identificadas. Esa labor asistencial no debe acabar luego del alta institucional, por lo que es fundamental brindar un seguimiento adecuado para conocer la evolución, en el corto y el largo plazo, tanto de los niños como de sus cuidadores. De esta manera, se podrá acompañar y ayudar o asistir en la toma de las conductas adecuadas a cada situación. A la vez, conocer la evolución permitirá valorar la calidad de la asistencia prestada y establecer un sistema de mejora en esta. Existen ejemplos exitosos de programas de seguimiento de recién nacidos en los países de las Américas y son progresivamente valorados, aunque sus características dependen de los recursos locales (3,4).

El Informe Mundial de Salud *¡Cada madre y cada niño contarán!* recomienda focalizar la atención en el período neonatal y pone de manifiesto la trascendencia de las acciones que permiten mantener la salud de los recién nacidos con mayor riesgo, luego de su inserción en la familia y en la sociedad. Por lo mencionado, el haber nacido con condiciones que pueden exponerlos a riesgos específicos hace necesario brindar seguimiento adecuado que contribuya a valorar la evolución del crecimiento y neurodesarrollo durante los primeros años de vida, al menos hasta el ingreso escolar (5). Algunas consecuencias vinculadas a las alteraciones perinatales podrán ser identificadas en forma inmediata al nacimiento o bien en el momento en que la función afectada debiera manifestarse; así, algunas secuelas pueden evidenciarse en los primeros días, semanas o meses de vida y, otras, en edades más tardías. Para ello, es esencial conocer cuáles son las alteraciones y los posibles daños con alta probabilidad de ocurrencia en esa población para monitorearlos de forma sistemática, de modo que se logre la detección temprana y el abordaje terapéutico y rehabilitación oportunos (6-9).

Por lo tanto, surge la necesidad de desarrollar una guía de seguimiento de los recién nacidos con condiciones de riesgo en América Latina y Caribe, con el fin de identificar y abordarlas de forma temprana y mejorar su salud y calidad de vida.

El objetivo de este trabajo es presentar una síntesis de evidencia de las recomendaciones de las *Directrices de práctica clínica basadas en la evidencia para el seguimiento de recién nacidos en riesgo,* una guía de práctica publicada por el Centro Latinoamericano de Perinatología/Salud de la Mujer y Reproductiva de la Organización Panamericana de la Salud en 2020, y aspectos de su implementación (10).

## MÉTODOS

### Objetivos y población diana considerada en la guía

Las *Directrices de práctica clínica informada en la evidencia para el seguimiento de recién nacidos en riesgo* (10), que sirvió de base para la elaboración de esta síntesis de evidencia, se desarrolló con el objetivo de brindar recomendaciones informadas en la evidencia para el seguimiento de los niños recién nacidos con condiciones de riesgo hasta los 2 años, con el fin de detectar y proveer cuidados específicos a condiciones que se presenten de forma temprana. La población diana está constituida por recién nacidos con condiciones de riesgo hasta los dos años (edad corregida para prematuros). Por recién nacidos en riesgo se entienden todos aquellos con una condición que necesita cuidado multidisciplinario después de su salida del proveedor de salud (prematuros y recién nacidos con alteraciones adquiridas o congénitas) o recién nacidos que parecen sanos y donde el seguimiento permitirá identificar tempranamente desenlaces anormales de salud.

### Metodología de elaboración de la guía

El documento que sirve de base a esta síntesis de evidencia siguió los métodos de desarrollo rápido de guías GRADE propuestos en el documento *Directriz para el fortalecimiento de los programas nacionales de guías informadas por la evidencia. Una herramienta para la adaptación e implementación de guías en las Américas,* publicado por la OPS en el 2018 (11) y en el *Manual para elaborar guías*, publicado por la Organización Mundial de la Salud en el 2016 (12). Se formó un grupo desarrollador multidisciplinario compuesto por expertos temáticos, epidemiólogos, salubristas y usuarios. No se identificó una guía susceptible a ser adaptada, por lo que se realizó la guía *de novo*. Se realizaron las búsquedas de la literatura hasta marzo del 2019 en bases de datos electrónicas (Pubmed, EMBASE, Cochrane, Epistemonikos), búsqueda manual y literatura gris. Luego, se crearon la síntesis y los perfiles de evidencia utilizando el enfoque GRADE (13). Se desarrolló un panel virtual compuesto de tres fases con expertos regionales con el fin de formular y graduar las recomendaciones. La primera fase correspondió a una encuesta donde se refinaron las recomendaciones preliminares, se evaluó el grado de acuerdo y se identificaron barreras y facilitadores; en una segunda fase virtual se revisaron las recomendaciones donde no se logró acuerdo y se revisaron los resultados de la encuesta; y en una tercera fase se dio el aval final. Las preferencias de pacientes fueron tomadas de la literatura y con la encuesta a una madre de un niño prematuro. Todos los participantes del panel y del grupo desarrollador firmaron un documento de conflicto de intereses, analizados por el equipo de coordinación de la guía. La guía fue desarrollada con el enfoque GRADE (*Grading of Recommendations Assessment, Development and Evaluation*), el cual permite formular las recomendaciones considerando la calidad de la evidencia, el balance entre el riesgo y el beneficio, los valores y preferencias de los pacientes, la aplicabilidad, los costos y, de forma global, el contexto de implementación (13). Se utilizó el enfoque DECIDE (14) (*Decisions and Practice based on Evidence*) fue utilizado para orientar las recomendaciones basado en la calidad de la evidencia, el efecto de las intervenciones, recursos, equidad, aceptabilidad y factibilidad (15). Los detalles metodológicos y la evidencia que apoya las recomendaciones están disponibles en la guía sobre la que se basa esta síntesis de evidencia (10).

### Alcance y usuarios de la guía

La guía que sirvió de base para la elaboración de esta síntesis de evidencia brinda recomendaciones informadas en la evidencia para el seguimiento de recién nacidos en riesgo hasta los dos años y corresponde a la primera fase de su seguimiento. Existe consenso en que el seguimiento durante los dos años permite identificar una gran cantidad de desenlaces anormales de funcionalidad neurológica, metabólica y motora. Las recomendaciones están dirigidas a todos los funcionarios del sector salud responsables de la atención primaria de los recién nacidos en riesgo: médicos generales, médicos familiares, pediatras, neonatólogos, oftalmólogos pediatras, otorrinolaringólogos pediatras, enfermeras y otros especialistas y personal multidisciplinario involucrado en la atención del recién nacido en riesgo. La guía pretende ser utilizada por tomadores de decisiones y miembros de entidades gubernamentales con el fin de facilitar el proceso de implementación. Cada país puede realizar la adaptación (11) de la guía según la normatividad, los recursos y la evidencia local para optimizar el seguimiento de recién nacidos en riesgo. La guía también contiene información para los padres, las madres y los cuidadores de recién nacidos en riesgo (10).

Los temas que se incluyen en la guía son: 1) criterios de egreso incluidas las pruebas de tamización; 2) información y apoyo para padres y cuidadores; 3) tamizaje durante la consulta de seguimiento; y 4) frecuencia del seguimiento de los niños en riesgo hasta dos años. No se incluyen aspectos relacionados con el manejo de enfermería ni comorbilidades.

### Cómo usar esta síntesis de evidencia

Para cada pregunta clínica se presenta un grupo de recomendaciones y buenas prácticas para el seguimiento de los recién nacidos en riesgo hasta los dos años.

En los cuadros 1 y 2 se presentan el nivel de calidad de la evidencia y la fuerza de la recomendación según el sistema GRADE, respectivamente.

## METODOLOGÍA DE DESARROLLO DE LA SÍNTESIS DE EVIDENCIA

Las *Directrices de práctica clínica basadas en la evidencia para el seguimiento de recién nacidos en riesgo* (10), una guía de práctica clínica desarrollada por el Centro Latinoamericano de Perinatología/Salud de la Mujer y Reproductiva de la OPS/OMS, tiene el fin de presentar las estrategias para el seguimiento de los recién nacidos con condiciones de riesgo desde su nacimiento hasta los 2 años, para avanzar hacia el logro de los Objetivos de Desarrollo Sostenibles (ODS). La información de la guía relacionada con la metodología, alcance, objetivos, resumen de las recomendaciones y la calidad de la evidencia se sintetizó empleando un formato predeterminado. Usando la estrategia de búsqueda de la guía y filtros para identificar estudios sobre consideraciones de implementación (15), se realizaron búsquedas de revisiones sistemáticas que abordaran aspectos de implementación (barreras, facilitadores, estrategias de implementación e indicadores). La estrategia incluyo los términos *adoption, uptake, utilization; taken implementation, dissemination, evidence-based treatment* y *barriers*. La búsqueda se realizó en Pubmed, Lilacs, Health Systems Evidence y Epistemonikos hasta enero del 2020. Asimismo, se revisaron los estudios primarios y reportes técnicos desarrollados en la Región; también se incluyeron las guías regionales y otros documentos de la OPS. No se evaluó la calidad de la evidencia incluida. Se seleccionaron revisiones sistemáticas y estudios primarios con el objetivo de identificar las consideraciones de implementación de las recomendaciones de la guía. Estas se organizaron de acuerdo con el tipo de barrera (recurso humano; recursos financieros, materiales y tecnológicos; conocimiento de la guía; y acceso). Para las barreras identificadas se seleccionaron los facilitadores y estrategias de implementación más efectivas según el contexto de la Región. A partir de la guía, se identificaron indicadores de proceso y de resultado de implementación. Por último, un grupo interdisciplinario de metodólogos y expertos temáticos de la OPS revisó los aspectos relacionados con la implementación. Además, y con base en el efecto de la pandemia de COVID-19 en la Región de las Américas, se elaboró, con el aporte del panel y los expertos de la OPS, un punto de buena práctica, no incluido en la guía, para optimizar la implementación de las recomendaciones en el contexto actual (16,17).

## RECOMENDACIONES

A continuación, se presentan las recomendaciones y los puntos de buena práctica que brindan orientación sobre el seguimiento del recién nacido en riesgo (10). Para cada pregunta clínica, se presenta el proceso de toma de decisiones para formular las recomendaciones de acuerdo con el enfoque GRADE (13).

**CUADRO 1.** Nivel de calidad de la evidencia según el sistema GRADE

Nivel de evidencia	Significado
**Alta ⊕⊕⊕⊕**	Es muy poco probable que nuevos estudios cambien la confianza que se tiene en el resultado estimado.
**Moderada ⊕⊕⊕Ο**	Es probable que nuevos estudios tengan un impacto importante en la confianza que se tiene en el resultado estimado y que estos puedan modificar el resultado.
**Baja ⊕⊕ΟΟ**	Es muy probable que nuevos estudios tengan un impacto importante en la confianza que se tiene en el resultado estimado y que estos puedan modificar el resultado.
**Muy baja ⊕ΟΟΟ**	Cualquier resultado estimado es muy incierto.

**CUADRO 2.** Fuerza de la recomendación y su significado según el sistema GRADE

Fuerza de la recomendación	Significado
**Fuerte**	Debe realizarse. Es poco probable que nueva evidencia modifique la recomendación. **SE RECOMIENDA HACERLO**
**Condicional**	Podría realizarse. Nueva evidencia podría modificar la recomendación. **SE SUGIERE HACERLO**
**Fuerte en contra**	No debe realizarse. Es poco probable que nueva evidencia modifique la recomendación. **SE RECOMIENDA NO HACERLO**
**Condicional en contra**	Puede no realizarse. Nueva evidencia podría modificar la recomendación. **SE SUGIERE NO HACERLO**
**√**	Punto de buena práctica

Pregunta 1. ¿Cuáles son los criterios para el egreso hospitalario de los recién nacidos de alto riesgo?	
Recomendaciones para niños prematuros	
N.°	Recomendación
1	Se recomienda que los recién nacidos con peso menor de 2500 g ingresen a un programa canguro (en modalidad continua o intermitente) o de la técnica de “contacto piel con piel” (antes de las 6 horas del nacimiento si no existe comorbilidad que lo contraindique) al egreso de la UCIN en instituciones o países donde se encuentre disponible, para aumentar la ganancia de peso y la promoción de la lactancia, así como disminuir el riesgo de muerte o de infección grave o sepsis. **Recomendación fuerte. Calidad de la evidencia: moderada ⊕⊕⊕Ο**
√	El programa madre canguro o la técnica de “contacto piel con piel” deben ser protocolizados y elaborados por equipos capacitados, que realizarán un monitoreo continuo que permita evaluar los resultados de los recién nacidos prematuros. Además, los padres deben recibir capacitación adecuada para el manejo del recién nacido en casa. **Punto de buena práctica**

Recomendaciones para recién nacidos en riesgo	
N.°	Recomendación
2	Se sugiere profilaxis con palivizumab para la prevención de episodios de bronquiolitis debido a virus sincicial respiratorio. Cada país debe seguir la recomendación de acuerdo con la indicación y la población diana para la cual fue aprobada. **Recomendación condicional. Calidad de la evidencia: muy baja ⊕ΟΟΟ**
√	Se sugiere se administre palivizumab en los recién nacidos con mayor vulnerabilidad. Cada país deberá tener sus recomendaciones locales de acuerdo con su epidemiología. La vacuna hexavalente, así como el resto de las vacunas, se deben aplicar según el calendario a la edad cronológica (aun estando internados en la UCIN). **Punto de buena práctica**
3	Se sugiere que se dé el egreso a los recién nacidos de alto riesgo con alimentación completa por vía oral y enteral (de preferencia por succión y, ocasionalmente, por sonda) bien sea con lactancia materna exclusiva, alimentación mixta, o sucedáneos de la leche materna y vigilando que hayan superado la hipoglucemia en casos en los que se haya presentado. **Recomendación condicional. Calidad de la evidencia: baja ⊕⊕ΟΟ**
√	Se debe vigilar que se aporte los volúmenes indicados y que la leche materna sea suficiente, ya que se puede presentar desnutrición asociada a la condición de alto riesgo que los aqueja y, por ende, ensombrecer el pronóstico tanto vital como neurosensorial. **Punto de buena práctica**
4	Se recomienda que se verifiquen los siguientes criterios de egreso de recién nacidos de alto riesgo en la UCIN, sumados a los criterios básicos que se realizan a todo recién nacido: **Recomendaciones para recién nacidos prematuros** Estabilidad clínica (temperatura de 36,5-37,5 °C) durante 24 horas para los recién nacidos prematuros con edades gestacionales de 35 semanas o más. Alimentación adecuada por vía oral para los recién nacidos en riesgo. En los recién nacidos prematuros menores de 34 semanas de edad gestacional, 48 horas de observación de alimentación oral completa son suficientes. Los prematuros deben tener buena succión al pecho materno y establecido el reflejo de succión-deglución-respiración, además de una adecuada estimulación por parte de la madre durante la alimentación. Se recomienda verificar si pueden regular su temperatura corporal en el contacto piel con piel. Ganancia de peso y crecimiento adecuados, bien sea una meta establecida de 18 g/kg/día de peso y 0,9 cm/semana de perímetro cefálico o que toleren una posición canguro prolongada sumada a una ganancia de peso de 15 g/kg/día hasta las 37 semanas y, a partir de la semana 38, de 8-11 g/kg/día. En recién nacidos prematuros, las metas de saturación de oxígeno deben ser mayores a 92 % y hasta 94%. **Recomendaciones para todos los recién nacidos en riesgo** Los recién nacidos deben poder mantener la posición supina antes del egreso. Se debe evaluar la ausencia de apneas en neonatos en riesgo que toleran la posición supina o prematuros en programa canguro por 48 horas luego de la suspensión del aporte de oxígeno. En recién nacidos con displasia broncopulmonar la meta de saturación de oxígeno es de 92%-95%. Registro de vacunación y el esquema ampliado de inmunizaciones para recién nacidos en la UCIN de acuerdo con el calendario local y a la edad real. Evaluación de riesgo social y familiar por trabajo social. **Recomendación fuerte. Calidad de la evidencia: baja ⊕⊕ΟΟ**
	Se recomienda el tamizaje de las siguientes condiciones antes del egreso de los recién nacidos, incluidos los de alto riesgo, según la legislación de cada país: Errores innatos del metabolismo y cardiopatías: las pruebas se deben realizar con muestra de gota seca obtenida por punción de talón, después de 24 a 36 horas de nacidos. El número y el tipo de enfermedades que se deben pesquisar depende del paquete de tamizaje de cada país. Enfermedad cardíaca congénita crítica: la evaluación se debe realizar con pulsoximetría después de 24 horas de nacimiento. Los recién nacidos que requirieron oxígeno suplementario deben ser tamizados después de 24 horas de estabilidad clínica en aire ambiente. Alteraciones neurológicas: la evaluación neurológica se debe realizar de forma completa y sistemática mediante el examen neurológico de Dubowitz, el de Amiel-Tison o los movimientos generales de Prechtel. Retinopatía de la prematuridad (ROP): se recomienda realizar tamizaje para detección de ROP en todo recién nacido con peso al nacer <2000 g y/o de 36 semanas o menos de EG con cualquier peso, que presente al menos una de las situaciones identificadas como factores de riesgo de ROP (18). Problemas auditivos: la evaluación se realiza con potenciales evocados auditivos automatizados (PEAA) o emisiones otoacústicas (EOA) antes del egreso. Si el recién nacido pasa más de cinco días en la UCIN, el tamizaje debe realizarse después de las 32 semanas de EG en un recién nacido estable y deben incluir PEAA. Anemia: al momento del egreso, se debe evaluar los niveles de ferritina y hierro corporal total y, con base en los resultados, administrar hierro a través de un complejo multivitamínico, un suplemento o fórmula materna fortificada. El plan de salida debe incluir monitorización de síntomas de osteopenia de la prematuridad y anemia, como taquicardia o pobre ganancia de peso, nivel de hemoglobina, la presencia de reticulocitosis y la ingesta adecuada de hierro en la dieta. Estabilidad de ganancia de peso de los niños en riesgo en la última semana antes del egreso. **Recomendación fuerte. Calidad de la evidencia: baja ⊕⊕ΟΟ**
√	Los resultados del tamizaje deben registrarse en la historia clínica y en la hoja de remisión, si es el caso. Si la entidad que recibe a un paciente remitido no puede certificar la realización del tamizaje, debe realizarlo antes del egreso del paciente. En general, si los recién nacidos no logran saturaciones de oxígeno >95%, deben ser evaluados por un cardiólogo con ecocardiografía. **Punto de buena práctica**
6	Se recomienda que los recién nacidos egresen de la UCIN con la primera cita asignada de control y el esquema de seguimiento. **Recomendación fuerte. Calidad de la evidencia: muy baja ⊕ΟΟΟ**
7	Se recomienda derivar a los recién nacidos en riesgo o prematuros al especialista o a interconsulta si se detecta alguna anomalía en el tamizaje antes del egreso. Para las cardiopatías congénitas, se recomienda que la derivación al especialista se realice lo antes posible, de acuerdo con la gravedad del cuadro clínico. **Recomendación fuerte. Calidad de la evidencia: muy baja ⊕ΟΟΟ**

Pregunta 2. ¿Qué información debe entregarse a los padres y cuidadores de los recién nacidos en riesgo al momento del egreso?	
N.°	Recomendación
8	Se recomienda que los padres reciban información sobre el manejo adecuado en casa del recién nacido en riesgo en el momento de salida de la UCIN neonatal, que incluye técnicas de secado de los recién nacidos después del baño, la técnica de contacto piel con piel, el cuidado de los ojos, el esquema de vacunación, la protección térmica, la lactancia materna, los requerimientos nutricionales, la técnica de reanimación cardiopulmonar, masajes, la interacción adecuada ente el padre y el niño, el plan de seguimiento, las posturas adecuadas, la administración de medicamentos o multivitamínicos en caso necesario, los signos de alarma y dónde acudir en caso de emergencia. **Recomendación fuerte. Calidad de la evidencia: baja ⊕⊕ΟΟ**
√	Se sugiere que se realicen talleres durante la hospitalización y no solo brindar la información al egreso, con el fin de asegurarse que los padres comprendan toda la información y no se presenten complicaciones por manejo inadecuado de los recién nacidos. Se debe entregar información impresa y en formato audiovisual para que los padres puedan consultar en casa. Esta información puede ser entregada de forma presencial, por correo electrónico y por mensajes de texto. **Punto de buena práctica**
9	Se sugiere que los padres de los niños en riesgo conozcan el daño producido por el tabaco y reciban información sobre las intervenciones para dejar de fumar con el fin de mejorar la calidad de vida de los recién nacidos. **Recomendación condicional. Calidad de la evidencia: baja ⊕⊕ΟΟ**
10	Se sugiere entregar a los padres participantes del programa madre canguro (en las instituciones donde se encuentre disponible) la información correspondiente para la atención en casa del recién nacido prematuro. **Recomendación condicional. Calidad de la evidencia: baja ⊕⊕ΟΟ**
√	Se debe entregar información a los padres sobre el esquema de seguimiento y explicar con claridad la importancia de cumplir con las visitas programadas. **Punto de buena práctica**

Pregunta 3. ¿Qué apoyo necesitan los padres y cuidadores de los recién nacidos en riesgo para disminuir la morbilidad y mortalidad neonatales?	
N.°	Recomendación
11	Se sugiere que se desarrollen programas de visita en casa a los recién nacidos en riesgo con el fin de mejorar el cuidado general, la interacción del padre y de la madre con el niño y la ansiedad de los padres en los países cuyo sistema de salud lo facilite. **Recomendación condicional. Calidad de la evidencia: muy baja ⊕ΟΟΟ**
√	El programa de visita en casa puede iniciar desde la UCIN o después del egreso. También puede realizarse en las instituciones prestadoras de servicios de salud o en grupos comunitarios. **Punto de buena práctica**
12	Se sugiere que los programas de seguimiento de recién nacidos de alto riesgo cuenten con servicios integrados de especialistas e interdisciplinas, que incluyan lugares de cuidado especial para estos niños. En su defecto, es ideal que cuenten con la derivación programada a los especialistas y servicio de ayuda social. **Recomendación condicional. Calidad de la evidencia: muy baja ⊕ΟΟΟ**
√	Los servicios para la atención de recién nacidos deben contar con: 1) promoción de la asistencia a los programas de seguimiento del recién nacido en riesgo, evaluaciones seriadas y diagnóstico oportuno; 2) evaluación psicosocial de los padres (psicóloga y trabajadora social); 3) talleres y material educativo sobre crianza, promoción del desarrollo, prevención de complicaciones neurosensoriales (estimulación temprana), prevención de infecciones respiratorias agudas (no fumar, vacunación al día) y 4) promover la asistencia a los programas de seguimiento para evaluaciones seriadas, diagnóstico y tratamiento oportuno. **Punto de buena práctica**

Pregunta 4. ¿Cuáles son las pruebas de tamizaje que deben realizarse durante el control de seguimiento del recién nacido en riesgo hasta los 2 años?	
N.°	Recomendación
13	Se recomienda realizar tamizaje para detección de ROP en todo recién nacido con peso al nacer <2000 g y/o de 36 semanas o menos de EG o con cualquier peso, que presente al menos una de las situaciones identificadas como factores de riesgo de ROP (18). **Recomendación Fuerte. Calidad de la evidencia: muy baja ⊕ΟΟΟ**
14	No se sugiere la evaluación con resonancia magnética cerebral o ecografía para el tamizaje de alteraciones del neurodesarrollo en niños de alto riesgo. Estas pruebas solo se realizarán en casos en los que se justifique. **Recomendación condicional en contra. Calidad de la evidencia: muy baja ⊕ΟΟΟ**
15	Se recomienda la evaluación neurológica con el examen de Dubowitz a una edad equivalente al término y con la prueba de Amiel-Tison, la escala de Bayley III (o posterior) o la prueba que se encuentre validada en cada país con el fin de tamizar alteraciones del neurodesarrollo en niños y niñas de alto riesgo. **Recomendación fuerte. Calidad de la evidencia: moderada ⊕⊕⊕Ο**
16	Se recomienda medir el perímetro cefálico durante la evaluación clínica sistemática del recién nacido en riesgo para la detección de hidrocefalia, utilizando la puntuación recomendada por la Organización Mundial de la Salud (19). **Recomendación fuerte. Calidad de la evidencia: baja ⊕⊕ΟΟ**
√	Se debe utilizar la misma escala en cada visita de seguimiento de los niños de alto riesgo. **Punto de buena práctica**
17	A todos los niños de alto riesgo, se recomienda la tamización de trastornos de la audición con emisiones otoacústicas evocadas transitorias (TEOAE) y respuesta auditiva automatizada de tallo (AABR, por su sigla en inglés). **Recomendación fuerte. Calidad de la evidencia: muy baja ⊕ΟΟΟ**
18	Se sugiere que el tamizaje de seguimiento de los niños en riesgo sea realizado por un equipo multidisciplinario compuesto por profesionales especializados con capacitación en el área según el nivel de complejidad y la disponibilidad; y conducido por el pediatra que realiza el seguimiento. **Recomendación condicional. Calidad de la evidencia: muy baja ⊕ΟΟΟ**
19	Se recomienda que derivar al especialista a los niños en riesgo antes de las 72 horas si se detecta alguna anomalía en el tamizaje. **Recomendación fuerte. Calidad de la evidencia: muy baja ⊕ΟΟΟ**
20	No se recomienda la tamización del espectro autista (M-CHAT, por su sigla inglés) de los niños en riesgo. **Calidad de la evidencia: muy baja ⊕ΟΟΟ**

Pregunta 5. ¿Cuál es el mejor esquema de seguimiento para los niños en riesgo menores de dos años?	
N.°	Recomendación
21	Se recomienda que los niños en riesgo tengan el siguiente esquema de seguimiento a dos años. **Recomendación fuerte. Calidad de la evidencia: muy baja ⊕ΟΟΟ (opinión de expertos)**

**Table:** 

Actividad	1 sem PE	2 sem PE	4 sem PE	1 m EC	2 m EC	3 m EC	4 m EC	5 m EC	6 m EC	8 m EC	9 m EC	10 m EC	12 m EC	14 m EC	15 m EC	16 m EC	18 m EC	20 m EC	22 m EC	24 m EC
**Controles clínicos:** Primer control entre los 3 y 5 días posteriores al egreso. Control quincenal si la ganancia de peso es normal. En caso contrario, control a los tres días o rehospitalización si hay pérdida de peso.	◉	•			◉		◉				◉		◉				◉			◉
**Evaluación neurológica detallada**				◉		◉			◉		◉		◉				◉			◉
**Ecografía cerebral**		•																		
**Evaluación del neurodesarrollo** Completa a los 24 meses					•		•		•				•		•					◉
**Evaluación auditiva** Potenciales evocados. Emisiones acústicas.				•		•			•				•		•					
					•											•				
**Evaluación oftalmológica** Retinopatía de la prematuridad	*	*	•			•				•										
	•																			
**Tamizaje de anemia (antes de los 12 meses) si el pediatra lo considera necesario**									•					◉						
**Inmunoprofilaxis** Tensión arterial. Ecocardiografía (último control luego del retiro de oxígeno).				**Esquema como el del recién nacido de término según el esquema de cada país**																
						◉														
					•		•				•									

Sem, semanas; m, meses; PE, posegreso; EC, edad corregida.

•: actividad; ◉: revisión detallada y completa.

**Figure:**
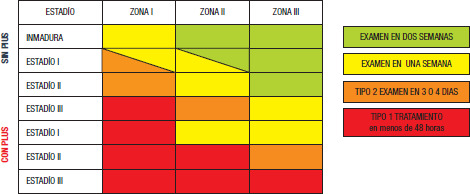


**Table:** 

N.°	Recomendación
√	Los niños en riesgo deben continuar con su esquema de seguimiento más allá de los dos años. **Punto de buena práctica**
√	Los niños deben tener seguimiento con su pediatra o neonatólogo de cabecera, y con un experto en seguimiento de niños de alto riesgo. **Punto de buena práctica**
√	Los niños en riesgo deben continuar su esquema de seguimiento en el contexto actual de la pandemia de la enfermedad por el nuevo coronavirus 2019 (COVID-19, por su sigla en inglés) con el cumplimiento de los protocolos institucionales y normatividad vigentes para mitigar el riesgo de contagio. Cuando el profesional, los padres y cuidadores lo consideren factible, se priorizará la atención remota por telesalud (p. ej., con seguimiento telefónico o con videollamadas). **Punto de buena práctica**

### Implementación

La guía en la que se basa esta síntesis de evidencia (10) recomienda que los siguientes actores apoyen la implementación de las recomendaciones: profesionales de la salud que atienden los niños recién nacidos prematuros en las salas de nacimiento y en las unidades de cuidado intensivo neonatal (pediatras, neonatólogos, fisiatras, kinesiólogos, enfermeras especializadas), sociedades científicas, entes gubernamentales, organizaciones no gubernamentales, instituciones universitarias, Organización Panamericana de la Salud, personal administrativo de instituciones con UCIN, y actores clave de los sistemas de salud de cada país. 

Dentro del proceso de implementación, es determinante identificar las posibles barreras, los facilitadores y las estrategias para mejorar la utilización de la guía. En el cuadro 3 se presentan algunos de estos elementos que pueden ser consideradas por los países (10,16,17,20-23).

En el recuadro 1 se sugieren los siguientes indicadores de pro-ceso y resultado de la implementación de la guía (10).

**CUADRO 3.** Barreras, facilitadores y estrategias de implementación relacionados con la guía informada en la evidencia para el seguimiento del recién nacido en riesgo

Aspecto	Barreras	Facilitadores	Estrategias de implementación
Recurso humano	Dificultad de contar con especialistas capacitados Falta de recurso humano para la realización adecuada de las pruebas de tamizaje	Proveedores de servicios de salud Instituciones educativas Entidades gubernamentales	Mejorar el entrenamiento de los profesionales en salud en las unidades de cuidado intensivo neonatal Incremento de la oferta de profesionales de la salud para cumplir con las recomendaciones de seguimiento de los recién nacidos en riesgo Para ayudar a la implementación de la técnica de piel con piel, los expertos sugieren contar con un cuarto cercano a las salas de parto y cesárea con el fin de brindar alojamiento conjunto y observación por 4 o 6 horas. En este período se observarían las principales complicaciones del recién nacido y de la madre en el período posparto inmediato
Conocimiento de la guía de práctica clínica	Los profesionales de la salud no conocen que existe una guía de seguimiento de recién nacidos en riesgo	Proveedores de servicios de salud Entidades gubernamentales Sociedades científicas	Socializar la guía a los profesionales de salud sobre dónde encontrarla guía en las instituciones Alojar la guía en las páginas web de los repositorios nacionales de guías: sitios web de entidades gubernamentales, sociedades científicas y hospitales Recordatorios en historias clínicas sistematizadas Difusión en revistas, boletines, aplicaciones móviles y páginas web
Recursos financieros, materiales y tecnológicos	No todos los países cuentan con los insumos o pruebas de tamizaje, lo cual puede afectar la salud de los recién nacidos en riesgo y no detectar las anomalías de manera oportuna	Entidades gubernamentales Proveedores de servicios de salud	Capacitaciones al personal de salud sobre las recomendaciones de la guía Disponibilidad de nuevas tecnologías recomendadas en la mayoría de las instituciones de salud Fortalecimiento de la normatividad para realizar las consultas de seguimiento Los gobiernos pueden promover políticas que garanticen el tamizaje obligatorio más completo para los recién nacidos en riesgo en todas las UCIN
Acceso	En zonas remotas a los centros de salud, se cuenta con poco acceso a especialistas para el tamizaje y seguimiento oportunos En el contexto actual de la pandemia de COVID-19, el seguimiento oportuno puede verse afectado Demora en las autorizaciones y trámites para recibir atención especializada Es posible que los padres no cuenten con el dinero para asistir a los controles de seguimiento, en especial, los provenientes de otras ciudades. No todas las instituciones cuentan con consulta de seguimiento de los recién nacidos, en especial en zonas rurales Desconocimiento de los padres sobre la importancia de la derivación al especialista	Entidades gubernamentales Proveedores de servicios de salud	Dotar a las UCIN con los insumos necesarios y con profesionales capacitados Mejora en los sistemas de referencia (derivación) y contrarreferencia regionales y nacionales Implementación del programa de visita en casa Fortalecimiento de protocolos institucionales y normatividad vigentes para mitigar el riesgo de contagio de COVID-19 Desarrollo de estrategias de telesalud (p. ej., con seguimiento telefónico y con videollamadas) Formulación o fortalecimiento de las políticas de atención a niños en riesgo Se debe contar con consultas de seguimiento como parte de la estrategia integral de manejo de los niños en riesgo. También se debe capacitar a los profesionales de la salud en la evaluación adecuada de esta población. Se debe capacitar a los padres sobre la importancia de asistir a las consultas de seguimiento
Sistema de salud	Falta de modelos de atención de cuidado neonatal que incluye a los recién nacidos en riesgo Falta de financiamiento y capacidad organizacional para proveer especialistas e insumos para el seguimiento adecuado	Entidades gubernamentales	Fortalecimiento de las políticas de atención de los recién nacidos, con énfasis en el tamizaje neonatal Buscar apoyo en redes nacionales e internacionales para la atención de calidad de los recién nacidos

**RECUADRO 1.** Indicadores de proceso y resultado en la implementación de las *Directrices de práctica clínica basadas en la evidencia para el seguimiento del recién nacido en riesgo*

Indicadores de proceso y resultado en la implementación de las *Directrices de práctica clínica basadas en la evidencia para el seguimiento de recién nacidos en riesgo* Proporción de recién nacidos en riesgo a quienes se realizó el tamizaje de errores innatos del metabolismo y cardiopatías antes del egreso hospitalario. Proporción de niños menores de 2 años que tuvieron por lo menos un tamizaje auditivo. Proporción de niños menores de 2 años en riesgo con derivación a un equipo de seguimiento, por zona geográfica. Proporción de oftalmólogos por habitante.

## CONCLUSIONES

La Organización Panamericana de la Salud pone a disposición de los gestores y del personal de la salud una síntesis sobre las recomendaciones informadas en la evidencia para el seguimiento oportuno y adecuado de los recién nacidos en riesgo. Asimismo, presenta aspectos a considerar como algunas barreras para la implementación de las recomendaciones, como la falta de modelos de atención nacionales, pruebas de tamizaje neonatal, personal capacitado, financiación de los sistemas de salud y un impacto negativo en el seguimiento oportuno en contexto actual de la pandemia de COVID-19. Se proponen como facilitadores el fortalecimiento de las políticas y programas de atención neonatal, mejorar la comunicación con los padres, las redes nacionales e internacionales y el desarrollo de estrategias de telesalud (p. ej., seguimiento por vía telefónica y videollamadas). Esperamos que esta síntesis favorezca la diseminación y el uso de las guías que elabora la OPS y contribuya a mejorar la calidad de la atención y la salud de los recién nacidos en la región de las Américas

## Agradecimientos.

Por el apoyo para la elaboración de esta síntesis de evidencia: Dra. Marcela Torres y Dr. Martín Alberto Ragusa, consultores del Departamento de Evidencia e Inteligencia para la Acción en Salud (EIH) de la OPS; Dr Pablo Durán, Asesor Regional en Salud Neonatal, Centro Latinoamericano de Perinatología, Salud de la Mujer y Reproductiva de la OPS, y al Dr. Ludovic Reveiz, Asesor del Departamento de Evidencia e Inteligencia para la Acción en Salud (EIH) de la OPS. Agradecemos los aportes por la revisión de los siguientes expertos: Dr. Andrés de Francisco Serpa, Director del Departamento de Familia, Promoción de la Salud y Curso de Vida de la OPS; Dra. Suzanne J. Serruya, Directora del Centro Latinoamericano de Perinatología, Salud de la Mujer y Reproductiva del OPS; Dr. Martín Cañón, médico y magíster en epidemiología clínica; Lucas Victor Alves, Instituto de Saúde Integral Prof. Fernando Figueira (IMIP) (Brasil); Dra. Alba Julietha Castro Gaona, Asociación Colombiana de Neonatología; Dra. Yesenia Castro Guillén, Hospital Gineco Obstétrico de Nueva Aurora Luz Elena Arismendi (Ecuador); Dra. Ana Lucía Díez, Hospital Roosevelt (Guatemala); Diana Fariña, Dirección Nacional de Maternidad e Infancia, Ministerio de Salud y Desarrollo Social (Argentina); Dra. Patricia Fernández, Dirección Nacional de Maternidad e Infancia, Ministerio de Salud y Desarrollo Social (Argentina); Dra. Florángel García, Servicio de Neonatología Hospital Central Universitario Antonio María Pineda, Consulta de Seguimiento (Venezuela); Andrea Ghione, Cátedra de Neonatología, Universidad de la República (Uruguay); Dra. Dania Hernández, Programa Nacional de Salud Reproductiva (Guatemala); Dr. Andrés A. Morilla Guzmán, Hospital Materno Infantil Dr. A. A. Aballí y jefe del Grupo Nacional de Neonatología (Cuba); Dra. Mónica Morgues, Departamento de Pediatría y Cirugía Infantil, Facultad de Medicina, Universidad de Chile; Dra. Fabiola López Olivan, Centro Nacional de Equidad de Género y Salud Reproductiva (México); Dra. Ana Quiroga, Dirección Nacional de Maternidad e Infancia, Ministerio de Salud y Desarrollo Social (Argentina); Dra. Diana Rodríguez, Hospital Italiano de Buenos Aires (Argentina); Dr. Juan Carlos Silva, Asesor Regional en Prevención de la Ceguera y Salud Ocular, OPS; Dra. Elina Yáñez, Hospital Docente Calderón (Ecuador); Dra. Roseli Calil, Consultora en Neonatología, Coordinación General de Salud Infantil y Lactancia Materna en la Secretaría de Atención de Salud, Ministerio de Salud (Brasil); Dra. Tatiana Cohimbra, consultora de la OPS (Brasil); Dra. Nathalie Charpak, Fundación Canguro (Colombia); Dra. Clara Esperanza Galvis Díaz, Hospital Militar Central, Coordinadora académica de la Asociación Colombiana de Neonatología; Dr. Arnoldo Grosman, Departamento de Salud Materno Infantil, Universidad Maimónides, Centro colaborador de la OPS (Argentina); y Dr. Eduardo Urman, Departamento de Salud Materno Infantil, Universidad Maimónides, Centro colaborador de la OPS (Argentina) y Dra. María Josefa Castro, Pediatra neonatóloga del Hospital Miguel Pérez Carreño (Venezuela).

## References

[1] Barradas DT, Wasserman MP, Daniel-Robinson L, Bruce MA, DiSantis KI, Navarro, FH, et al. . Hospital utilization and costs among preterm infants by payer: nationwide inpatient sample, 2009. Matern Child Health J. 2016;20(4):808-18. Doi: 10.1007/s10995-015-1911-y. 10.1007/s10995-015-1911-y PMC4794344 26740227

[2] Brumana L, Arroyo A, Schwalbe NR, et al. Maternal and child health services and an integrated, life-cycle approach to the prevention of non-communicable diseases. BMJ Glob Health 2017;2:e000295. doi:10.1136/bmjgh-2017-000295. 10.1136/bmjgh-2017-000295 PMC5656183 29082005

[3] Rite S, Peres A, Sanz E, et al. Criteria for hospital discharge of the healthy term newborn after delivery. Anales de Pediatria. 2017;86(5):289. 10.1016/j.anpedi.2016.08.011 27746077

[4] Teune MJ, Bakhuizen S, Gyamfi Bannerman C, Opmeer BC, van Kaam AH, van Wassenaer AG, et al. A systematic review of severe morbidity in infants born late preterm. Am J Obstet Gynecol. 2011;205(4):374.e1-9. Doi: 10.1016/j.ajog.2011.07.015. 10.1016/j.ajog.2011.07.015 21864824

[5] Wehrmeister FC, Ferreira LZ, et al. Are the poorest poor being left behind? Estimating global inequalities in reproductive, maternal, newborn and child health. BMJ Global Health 2020;5:e002229. doi:10.1136/bmjgh-2019-002229. 10.1136/bmjgh-2019-002229 PMC7042578 32133180

[6] Orton J, McGinley JL, Fox LM, Spittle AJ. Challenges of neurodevelopmental follow-up for extremely preterm infants at two years. Early Hum Dev. 2015;91(12):689-94. Disponible en: http://www.embase.com/search/results?subaction=viewrecord&from=export&id=L607449004 10.1016/j.earlhumdev.2015.09.012 26513630

[7] Marlow N. Neurocognitive outcome after very preterm birth. Arch Dis Child Fetal Neonatal Ed. 2004;89(3):F224-8. 10.1136/adc.2002.019752 PMC1721665 15102725

[8] Serenius F, Kallen K, Blennow M, Ewald U, Fellman V, Holmstrom G, et al. Neurodevelopmental outcome in extremely preterm infants at 2.5 years after active perinatal care in Sweden. JAMA. 2013;309:1810-20. 10.1001/jama.2013.3786 23632725

[9] Organización Mundial de la Salud.Survive and thrive: transforming care for every small and sick newborn. Key findings. Ginebra: OMS; 2018. Disponible en: https://apps.who.int/iris/bitstream/handle/10665/276655/WHO-FWC-MCA-18.11-eng.pdf?ua=1 (Mayo 2021)

[10] Organización Panamericana de la Salud. Directrices de práctica clínica basadas en la evidencia para el seguimiento de recién nacidos en riesgo. Versión abreviada. Washington, D.C.: OPS; 2020. Disponible en: https://iris.paho.org/bitstream/handle/10665.2/52903/OPSFPLCLP200017_spa.pdf?sequence=7&isAllowed=y Acceso en mayo del 2021.

[11] Organización Panamericana de la Salud. Directriz para el fortalecimiento de los programas nacionales de guías informadas por la evidencia. Una herramienta para la adaptación e implementación de guías en las américas. Washington, D.C.: OPS; 2018. Disponible en: http://iris.paho.org/xmlui/handle/123456789/49145. Acceso en junio del 2019.

[12] Organización Mundial de la Salud. Handbook for guideline development (2° ed.). Ginebra: OMS; 2014. Disponible en: https://www.who.int/publications/guidelines/handbook_2nd_ed.pdf?ua=1 Acceso en junio del 2016.

[13] Guyatt GH, Oxman AD, Kunz R, Atkins D, Brozek J, Vist G. et al. GRADE guidelines: 2. Framing the question and deciding on important outcomes. J Clin Epidemiol. 2011;64(4):395-400. 10.1016/j.jclinepi.2010.09.012 21194891

[14] DECIDE (2011-2015) [Internet]. Disponible en: http://www.decide-collaboration.eu/evidence-decision-etd-framework Acceso en agosto del 2019.

[15] Lewis CC, Fischer S, Weiner BJ, et al. Outcomes for implementation science: an enhanced systematic review of instruments using evidence-based rating criteria. Implementation Sci. 2015;10:155. Doi: 10.1186/s13012-015-0342-x 10.1186/s13012-015-0342-x PMC4634818 26537706

[16] Duran P, Sommer JA, Otero P, Daus M, Benitez S, Serruya S, et al. Information and communication technologies in neonatal health and evidence-based interventions. Rev Panam Salud Publica. 2020;44:e123. Doi: 10.26633/RPSP.2020.123 PMC7655061 33196698

[17] Organización Panamericana de la Salud. Consideraciones para el fortalecimiento del primer nivel de atención en el manejo de la pandemia de COVID-19. Washington D.C.: OPS; 2020. Disponible en: https://iris.paho.org/bitstream/handle/10665.2/53112/OPSIMSHSSCOVID-19200035_spa.pdf?sequence=1&isAllowed=y Acceso en mayo del 2021.

[18] Organización Panamericana de la Salud. Guía de práctica clínica para el manejo de la retinopatía de la prematuridad. Washington, D.C.: OPS; 2018. Disponible en: https://iris.paho.org/handle/10665.2/34948

[19] Organización Mundial de la Salud. Child growth standards. Disponible en: https://www.who.int/tools/child-growth-standards/

[20] Nair M, Yoshida S, Lambrechts T, Boschi-Pinto C, Bose K, Mason EM, et al. Facilitators and barriers to quality of care in maternal, newborn and child health: a global situational analysis through metareview. BMJ Open. 2014;4(5):e004749. Doi: 10.1136/bmjopen-2013-004749 PMC4039842 24852300

[21] Chan G, Bergelson I, Smith ER, Skotnes T, Wall S. Barriers and enablers of kangaroo mother care implementation from a health systems perspective: a systematic review. Health Policy and Planning. 2017;32(10):1466-1475. Doi: 10.1093/heapol/czx098 PMC5886293 28973515

[22] Lassi ZS, Middleton P, Bhutta ZA, Crowther C. Health care seeking for maternal and newborn illnesses in low- and middle-income countries: a systematic review of observational and qualitative studies. F1000Research. 2019;8:200. Doi: 10.12688/f1000research.17828.1 PMC6480947 31069067

[23] Clark C, Maher L, Rudy M, Pitoniak J, Watling R, Tanta K. Facilitators and barriers to neonatal intensive care unit follow-up program attendance: a critically appraised topic. Journal of Occupational Therapy, Schools, & Early Intervention. 2018;11(2):109-123.

